# High incidence of fosfomycin-resistant uropathogenic *E. coli* among children

**DOI:** 10.1186/s12879-023-08449-9

**Published:** 2023-07-17

**Authors:** Wedad M. Abdelraheem, W. K.M Mahdi, Ibtehal S. Abuelela, Noha Anwar Hassuna

**Affiliations:** 1 Medical Microbiology and Immunology Department, Faculty of Medicine, Minia University, Minia, Egypt; 2 Pediatric Department, Faculty of Medicine, Minia University, Minia, Egypt

**Keywords:** Fosfomycin resistance, *Escherichia coli*, Urinary tract infections, Phylogenetic analysis

## Abstract

**Background:**

There are few epidemiological or molecular data on *Escherichia coli (E. coli*) strains resistant to fosfomycin. In this study, we described the occurrence and characterization of fosfomycin-resistant uropathogenic *E. coli* (UPEC) isolated from children.

**Materials and methods:**

This study was carried out on 96 *E. coli* isolates obtained from children with urinary tract infections. Two methods were performed to detect fosfomycin resistance: The agar dilution method and the rapid fosfomycin test. The disc diffusion method was done to detect the antimicrobial susceptibility pattern of all isolates. The phylogenetic grouping of all isolates was done according to the modified Clermont method. Conventional PCR was performed to detect plasmid-mediated fosfomycin-resistant genes (*fos* genes) and the *bla*_CTX−M_ gene.

**Results:**

Analyses of data were performed by SPSS software. A high percentage of fosfomycin resistance (37/96; 38.5%) was reported among UPEC isolates. The fosfomycin-resistant strains showed a higher resistance rate than fosfomycin-susceptible isolates to different antibiotics. E group (62.2%) was the most predominant phylogenetic group among the fosfomycin-resistant UPEC isolates, followed by Group B2 (21.6%) and group D (13.5%). The *fos* genes were detected in 21 isolates with *the fos*A3 gene as the most frequent, which was detected in 11 isolates followed by *fos*A (8), *fos*C2 (4), *fos*A4(1), and *fos*A5(1) genes.

**Conclusion:**

This is the first report of a high prevalence of plasmid-mediated fosfomycin-resistant UPEC in Egypt. All of these isolates were multidrug-resistant to the tested antibiotics. Close monitoring of such strains is mandatory to prevent widespread dissemination of the genes code for antibiotic resistance.

## Introduction

Urinary Tract Infection (UTI) is the most common bacterial infection between children with *E. coli* is the most common cause. It was reported that 8% of girls and 2% of boys are affected by UTI at the age of 7 years [1]. Early diagnosis and treatment of UTI is important as there is increased risk of renal disease in children below the age of 5 years which result of morbidity. Uropathogenic *E. coli* (UPEC) with different phylogenetic characteristics and virulence profiles are multiple drug resistant (MDR) isolates which make them a serious, challenging health problem including those producing extended-spectrum β-lactamases (ESBLs) and/or carbapenemases, resulting in major limitations on the therapeutic options available to treat urinary tract infections (UTIs) [2, 3]. Fosfomycin is an old antibiotic that has reemerged to overcome antibiotic resistance and is also commonly used as a first-line oral agent for uncomplicated UTI [4]. Recently a review of fosfomycin use in children recommends it for the treatment of UTI and osteomyelitis, especially for multidrug-resistant bacteria [5]. Fosfomycin prevents the initial step of peptidoglycan synthesis through the inactivation of N-acetyl glucosamine transferase (MurA), which prevents the formation of N-acetylmuramic acid from N-acetylglucosamine and phosphoenol pyruvate. Fosfomycin crosses the bacterial cell wall using two transport systems; glycerol phosphate (GlpT) and hexose phosphate (UhpT). Fosfomycin resistance can result from mutations in the chromosomal glpT and uhpT genes or amino acid mutations in the active site of the MurA target. The production of plasmid-encoded fosfomycin-inactivating enzymes (*fos* genes) is another important mechanism for fosfomycin resistance. Different fosfomycin-modifying enzymes have been described: FosA, FosB, FosC, and FosX, encoded by the *fos* gene, which inactivates fosfomycin by cleaving the oxirane ring. FosA enzymes are the most frequently reported fosfomycin-modifying enzymes and are common in Enterobacteriaceae. The FosA3 enzyme is the most commonly identified FosA determinant in *E. coli* [6]. Other *fos* genes, such as *fosA4, fosA5*, and *fosA6* have also been less frequently identified in *E. coli* [7]. Therefore, this study aimed to investigate the prevalence of fosfomycin-resistant *E. coli* isolated from children with UTIs in general, then to determine the distribution of the *fos* genes in these isolates. To the best of our knowledge, there are few epidemiological and molecular data regarding the phylogenetic grouping of fosfomycin-resistant *E. coli* [8, 9]. Accordingly, we aimed to evaluate the frequency of fosfomycin resistance and phylo-group fosfomycin-resistant and fosfomycin-susceptible isolates.

## Patients and methods

In this cross sectional descriptive study which has been done during the period from October 2021 till March 2022, all children aged from 2–16 years old complaining of UTI manifestations were selected from outpatient clinic - Minia university hospital. Exclusion criteria include: A child with a catheter associated UTI, A child diagnosed with congenital anomalies, Previous surgery of the genitourinary tract (except circumcision in male children), A child has history of chronic renal diseases or found an immune compromising agents as HIV, malignancy and chronic use of corticosteroids or other immunosuppressive drugs. All the included children in the study were exposed to the following: detailed history taking with special focus on the presence of urological manifestations (dysuria, loin pain, frequency, supra-pubic pain, change of the color of urine, enuresis), treatment with certain drugs for long period, past history of renal diseases or family history of renal diseases and full clinical examination.

The number of patients required in this study was estimated using the following equation[10]:

1 $$Z_{1 - \alpha /2}^2P(1 - P)/{d^2}$$

*Z*_1−*α*/2_ = *is standard normal variate* (*at 5% type 1 error* (*P* < 0.05) it is 1.96.

*P* = Expected proportion in population. d = Absolute error or precision.

### Isolation of *E. coli* from urine samples

A total of 241 midstream urine samples were collected from children (2–16 years old) attending the Pediatric outpatient clinics of Minia University hospital. Urine samples were transported to the laboratory for processing within 2 h of collection. Positive cultures were identified by detection of ≥ 10^5^ CFU/ml. *E. coli* identification was done by inoculation of urine samples into MacConkey and Eosin Methylene Blue agar (Oxoid, UK). Further identification was done by Gram staining and biochemical reactions of the suspected colonies [10].

### Phenotypic detection of fosfomycin-resistant *E. coli* strains

#### Agar dilution method

According to CLSI guidelines, the only approved method for testing fosfomycin MIC is agar dilution using agar media supplemented with 25 µg/mL of glucose-6-phosphate [11]. Commercial fosfomycin powder (Zambon, S.P.A), glucose-6-p powder (Oxoid, England), and Muller Hinton agar (Oxoid, England) were used. Briefly bacterial suspension of 0.5McFarland turbidity was prepared. Fosfomycin powder was diluted to obtain concentrations ranging from 2 to 1024 µg/ml. Mueller Hinton agar supplemented with 25 µg/mL of glucose-6-phosphate was dissolved in distilled water and then autoclaved. One part of the antimicrobial solution to nine parts of molten agar in every petri dish was added. Two µl of each isolate was spotted off the agar starting from the lowest antibiotic concentration. Two plates were prepared, one containing bacterial suspension with no antimicrobial solution as a growth control and the other containing plain media only as purity plates. The plates were inverted and incubated for 24 h at 37 ºC. Then MICs were recorded as the lowest concentration shows no visible growth on the inoculum spot.

#### Rapid fosfomycin NP test

The rapid fosfomycin NP test was developed to detect fosfomycin resistance in *E. coli* isolates and its results are obtained in 1 h 30 min. The test is based on glucose utilization and the detection of bacterial growth in the presence of fosfomycin. Bacterial growth is visually detected by an orange-to-yellow color change of red phenol, a pH indicator. The rapid fosfomycin NP test was done according to Nordmann’s instructions as follows: Briefly, the rapid fosfomycin NP solution was prepared by mixing the culture medium, glucose, pH indicator, and distilled water. In a 96-well polystyrene micro test plate 50 µl of bacterial suspension (3 McFakrland standard) was added to two wells, the first containing 150 µl of rapid fosfomycin NP solution without Fosfomycin (Positive control), and the second containing 150 µl of rapid fosfomycin NP solution supplemented with 25 µg/ml glucose-6-phosphate and 40 µg/ml fosfomycin. The same procedure was performed using 50 µl Sodium Chloride instead of the bacterial suspension, as negative controls. The plate was incubated at 37 ºC in an aerobic incubator and the micro-test plates were visually inspected every 30 min [12].

### Antibiotic susceptibility pattern of *E. coli* isolates

Antimicrobial susceptibility testing for all *E. coli* isolates to the following antibiotics: Cefazolin, Ampicillin-Sulbactam, Cefoxitin, Ceftazidime, Levofloxacin, Nitrofurantoin, Tetracycline, and Meropenem were determined by the disk diffusion method according to the instructions of the Clinical and Laboratory Standards Institute (CLSI) [11].

### Phenotypic detection of plasmid-mediated fosfomycin-resistant *E. coli* by phosphonoformate test

Plasmid-encoded fosfomycin-inactivating enzymes (Fos enzymes) were detected by a modified Kirby-Bauer disk diffusion test. In brief, a Mueller-Hinton agar plate was inoculated with a 0.5 McFarland suspension of the isolate assayed. Two disks were placed on the agar: a 200-µg fosfomycin disk, and a disk with both 200-µg fosfomycin and 100-µg phosphonoformate. The diameter of the inhibition zone around each disk was measured after 24 h of incubation at 37 °C. The Fos enzyme-mediated fosfomycin resistance is inhibited by phosphonoformate and is demonstrated by an increase in the diameter of the growth inhibition zone by ≥ 5 mm [13].

### Molecular characterization

#### DNA extraction

DNA extraction for all *E. coli* isolates was done by boiling method as follow: After overnight broth culture for each isolate, 1.5 mL of bacterial broth was loaded into sterile microtubes. The tubes were centrifuged at 5,000 × *g* for 3 min by microtubes centrifuge (Hamburg, Germany). The supernatant was eliminated, and the pellet was suspended in 200 µl molecular biology-grade water and mixed by pipetting. The tubes were subjected to boiling at 100 °C in a water bath for 14 min then; sudden cooling in ice for 20 min, followed by centrifugation for 3 min at 5,000 × *g*. The supernatant containing DNA was transferred to a newly labeled tube for PCR study.

#### Phylogenetic grouping of *E. coli* isolates

Phylogenetic grouping was done according to the new Clermont method [14]: A multiplex PCR was used to group the *E. coli* isolates based on the presence or absence of 4 genes: *arpA, chuA, yjaA*, and *TspE4*. This method was used to classify *E. coli* isolates into 8 phylogroups (A, B1, B2, C, D, E, F, and cryptic clade). Multiplex PCR was carried out in a 25 µL reaction mixture, including 10 µl of hot start 2x Taq DNA Polymerase Master Mix (Bioline, USA), 5 µL nuclease-free water, 1 µL (10 µM), each primer, and 2 µL (100 ng/µl), template DNA. Amplification was carried out as follows: initial denaturation at 94 C° (10 min), 30 cycles of denaturation at 94 °C (30 s), annealing at 59 °C (30 s), and elongation at 72 °C (60 s), followed by a final extension at 72 °C (5 min). The PCR products were analyzed by running the PCR product through 1% agarose in TBE buffer at 90 V and visualization under UV trans-illumination.

#### Detection of ***fos*** genes and ***bla***_CTX-M_ gene

Conventional PCR was performed to detect *fos* genes and CTX-M gene in Fosfomycin-resistant isolates using primers and conditions listed in Table 1 [13, 15–18]. The amplification reactions were carried out in 25 µl volumes containing 12.5 µl of PCR master mix (Bioline, USA Inc.), 1 µl of each primer (10 pmol/µl), 8.5µL nuclease-free water, and 2 µl of 100 ng/µl template DNA.

**Table 1:** PCR Primers and conditions of the studied genes

Gene	Primer sequence	Annealing temp	Product size(bp)
*arp*A	F-AACGCTATTCGCCAGCTTGC R-TCTCCCCATACCGTACGCTA	59 °C	400
*chu*A	F-ATGGTACCGGACGAACCAAC R-TGCCGCCAGTACCAAAGACA		288
*yja*A	F-CAAACGTGAAGTGTCAGGAG R-AATGCGTTCCTCAACCTGTG		211
*tsp*E4C2	F-CACTATTCGTAAGGTCATCC R-AGTTTATCGCTGCGGGTCGC		152
*fos*A	F-ATCTGTGGGTCTGCCTGTCGT R-ATGCCCGCATAGGGCTTCT-3’	59.5 °C	271
*fos*A3	F- CCTGGCATTTTATCAGCAGT R- CGGTTATCTTTCCATACCTCAG	57.5 °C	221
*fos*A4	F-CTG GCG TTT TAT CAG CGG TT R -CTT CGC TGC GGT TGT CTT T	60 °C	234
*fos*A5	F-TATTAGCGAAGCCGATTTTGCT R-CCCCTTATACGGCTGCTCG	55 °C	177
*fos*A6	F- GCTACGGTTCAGCTTCCAGA R- CGAGCGTGGCGTTTTATCAG	58 °C	242
*fos*B	F-ATATGATCAAAGGAATAAATC R-CATATGAAAATTCATATGAGG	40 °C	767
*fos*C2	F-TGGAGGCTACTTGGATTTG R-AGGCTACCGCTATGGATTT	50.5 °C	217
*bla*_CTX−M_	F-SCS ATG TGC AGY ACC AGT AA R-CCG CRA TAT GRT TGG TGG TG	57 °C	554

### Statistical analysis

Analyses of data were performed by SPSS software version 23 (SPSS Inc., Chicago, IL, USA). Categorical data are presented as number and percentage. The Chi-square test was used to determine the significant differences. The odds ratios and corresponding p-values for the samples were analyzed by logistic regression analysis. A two-tailed p-value of ˂ 0.05 was considered statistically significant.

## Results

### Clinical characteristics of children with UTI

A total of 241 children with UTI between 2 and 16 years were identified. Of these, 126 (52.2%) patients had a first episode of UTI and 115 (47.7%) had recurrent UTI. The median age at presentation was 6 years old, females were predominant (N = 151, 62.7%). Other risk factors such as, history of prolonged antibiotics administration (N = 190, 78.8%), Family history of UTI (N = 174, 72.2%) and enuresis (N = 153, 63.5%) were predominant among children with UTI.

### Isolation of *E. coli* from urine samples

Out of 241 urine samples obtained from children suffering from UTIs, 170 isolates were confirmed as positive cultures. 96 of these cultures were confirmed to be *E. coli* (56.5%).

### Phenotypic detection of fosfomycin-resistant *E. coli* strains

Two methods were performed to detect fosfomycin resistance: The agar dilution method and the rapid fosfomycin test. Out of 96 *E. coli* isolates, 37 (38.5%) were fosfomycin resistant (MIC ≥ 256 µg/ml) by agar dilution method while, 36 (37.5%) *E. coli* isolates were reported as fosfomycin resistant by the rapid NP test. There was a good correlation between the agar dilution susceptibility test and the rapid fosfomycin NP test findings (97.3% sensitivity and 100% specificity), both for susceptible and resistant isolates. All but one fosfomycin-resistant isolates detected by agar dilution method converted to yellow color (resistant) by the rapid fosfomycin NP test.

### Antibiotic susceptibility pattern of *E. coli* isolates

The antimicrobial resistance rates stratified by fosfomycin resistance or susceptibility categories are summarized in Table 2. The fosfomycin-resistant strains studied in this work showed a higher resistance rate than fosfomycin-susceptible isolates to different antibiotics with significant differences to cefazolin, cefoxitin, ceftazidime (*p*-value < 0.05). Logistic regression analysis identified history of prolonged antibiotics administration and history of recurrent UTI as independent risk factors for fosfomycin resistance and MDR as shown in Table 3 (OR ˃ 1 and P ˂0.05). Also as will be mentioned below, UPEC phylogenetic group E was found to be an important risk factor for fosfomycin resistance (OR = 5.296, 95% CI: 1.667–10.167, P value = 0.001).

**Table 2:** Antimicrobial resistance pattern of *E. coli* isolates stratified by fosfomycin resistance or susceptibility categories

Antibiotic resistance		Fos R isolates N = 37(%)	Fos S isolates N = 59(%)	P-value
	**Cefazolin**	37 (100%)	52 (88.1%)	0.03*
	**Ampicillin-Sulbactam**	26 (70.3%)	29 (49.2%)	0.11
	**Cefoxitin**	30 (81.1%)	31 (52.5%)	0.01*
	**Ceftazidime**	35 (94.6%)	43 (72.9%)	0.02*
	**Levofloxacin**	34 (91.9%)	44 (74.6%)	0.07
	**Nitrofurantoin**	24 (64.8%)	49 (83.1%)	0.23
	**Tetracycline**	33 (89.2%)	46 (78%)	0.16
	**Meropenem**	19 (51.4%)	19 (32.2%)	0.17
	**MDR**	30 (81.1%)	39(66.1%)	0.03*

Fos R, Fosfomycin resistant isolates; Fos S Fosfomycin susceptible isolates; * significant

**Table 3:** Logistic regression analysis of risk factors for MDR and fosfomycin resistance in children with UTI;2

Risk factor	Resistance	Odds ratio	95% CI	P- value
**History of prolonged antibiotics administration**	MDR	5.796	1.757–10.436	0.000*
	Fos resistance	2.427	0.887–3.142	0.003*
**History of recurrent UTI**	MDR	2.169	0.774–2.347	0.009*
	Fos resistance	4.026	1.393–6.671	0.002*
**Phylogenetic group E**	MDR	1.642	0.496- 0.800	0.161
	Fos resistance	5.296	1.667–10.167	0.001*

Fos R, Fosfomycin resistance; MDR, multi drug resistance; * significant

### Phylogrouping of UPEC isolates

A total of 91 of 96 UPEC isolates were assigned to 3 phylogenetic groups (B2, D, and E) using the quadruplex PCR method. The remaining 5 isolates were categorized as an unknown group. Group B2 was the predominant phylogenetic group in the fosfomycin-susceptible isolates, 32/59 (54.2%). On the other hand, group E was the predominant phylogenetic group in the fosfomycin-resistant isolates, 23/37 (62.2%) (Table 4).

**Table 4:** The phylogenetic groups of *E. coli* isolates stratified by fosfomycin resistance or susceptibility categories:

Phylo-group	Fos R isolates N = 37(%)	Fos S isolates N = 59(%)	P-value
B2 *(arpA*^*−*^, *chuA*^*+*^, *yjaA*^*+*^,*TspE4*^*+*^)	8 (21.6%)	32(54.2%)	0.00*
D (*arpA*^*+*^, *chuA*^*+*^, *yjaA*^*−*^,*TspE4*^*−*^)	5(13.5%)	14(23.7%)	
E (*arpA*^*+*^, *chuA*^*+*^, *yjaA*^*+*^, *TspE4*^*−*^, *E*^*+*^)	23 (62.2%)	9(15.3%)	
Unknown (*arpA*^*+*^, *chuA*^*+*^, *yjaA*^*+*^, *TspE4*^*+*^*)*	1(2.7%)	4(6.8%)	

Fos R, Fosfomycin resistant isolates; Fos S Fosfomycin susceptible isolates; * significant

### Phenotypic and genotypic characterization of plasmid-mediated fosfomycin resistant isolates

**Fig. 1:**
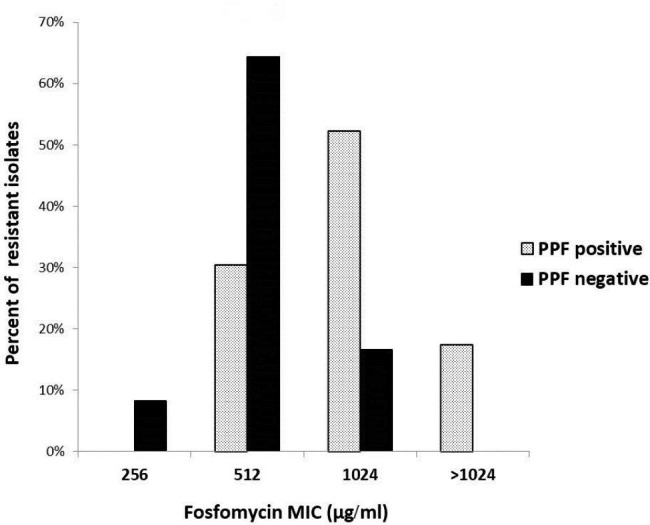
Fosfomycin MIC of the phosphonoformate test positive and negative isolates: PPF, phosphonoformate test

Phosphonoformate test was performed to detect plasmid-encoded fosfomycin-inactivating enzymes. Out of 37 fosfomycin resistant, *E. coli* isolates, 23 (62.2%) were phosphonoformate test positive. The phosphonoformate test positive isolates showed a higher fosfomycin MIC than the phosphonoformate test negative isolates with the median MIC of 1024 µg/ml for the phosphonoformate test positive isolates versus 512 µg/ml for the phosphonoformate test negative isolates (Fig. 1). PCR was performed to detect the plasmid-mediated fosfomycin-resistant genes in the fosfomycin-resistant isolates. The *fos* genes were detected in 21 isolates with 4 isolates harbor 2 genes of them. All of these isolates were phosphonoformate test positive but 2 phosphonoformate test positive isolates didn’t harbor any of the tested *fos* genes. Antibiotic-resistant pattern and molecular characterization of the 23 plasmid-mediated fosfomycin-resistant isolates were listed in Table 5. The predominant fosfomycin-resistant gene in this study was *fosA3*, which was found in 11 isolates (47.8%), followed by *fos*A, *fos*C2, *fos*A4, *and fos*A5 genes which were detected in 8(34.8%), 4(17.4%), 1(4.3%), and 1(4.3%) of the 23 phosphonoformate test positive isolates, respectively. All but 3 of the 11 *fos*A3-positive isolates in our study were also CTX-M gene positive. However, the *fos*A6 and *fos*B genes weren’t detected in any of the isolates (Table 6).

**Table 5:** Antibiotic resistance pattern and molecular characterization of the plasmid mediated fosfomycin-resistant isolates

UPEC ID	group	Resistance phenotype	genetic profile
1,14,33	E	MEM, LEV, TE, CZ, CAZ, FOX	*arpA*^*+*^, *chuA*^*+*^, *yjaA*^*+*^, *E, FosA*^*+*^
16, 42	E	MEM, LEV, TE, CZ, CAZ, N, FOX	*arpA*^*+*^, *chuA*^*+*^, *yjaA*^*+*^, *E, FosA*^*+*^, *FosC2*^*+*^
58	E	LEV, TE, CZ, CAZ, N,	*arpA+, chuA*^*+*^, *yjaA*^*+*^, *E, FosA*^*+*^, *FosA5*^*+*^
30	E	LEV, CZ, CAZ, N, FOX	*arpA*^*+*^, *chuA*^*+*^, *yjaA*^*+*^, *E, FosA*^*+*^, *FosA4*^*+*^
81,93	E	LEV, TE, CZ, CAZ, N,A/S, FOX	*arpA*^*+*^, *chuA*^*+*^, *yjaA*^*+*^, *E, FosA3*^*+*^
21, 44,75	E	MEM, LEV, TE, CZ, CAZ, N, A/S, FOX	*arpA*^*+*^, *chuA*^*+*^, *yjaA*^*+*^, *E, FosA3*^*+*^, *CTX-M*^*+*^
17,62, 77	E	LEV, TE, CZ, CAZ, N, A/S, FOX	*arpA*^*+*^, *chuA*^*+*^, *yjaA*^*+*^, *E, FosA3*^*+*^, *CTX-M*^*+*^
89	E	LEV, TE, CZ, CAZ, N, FOX	*arpA*^*+*^, *chuA*^*+*^, *yjaA*^*+*^, *E*,
12	E	LEV, TE, CZ, CAZ, N, FOX	*arpA*^*+*^, *chuA*^*+*^, *yjaA*^*+*^, *E, CTX-M*^*+*^
29	B2	LEV, TE, CZ, CAZ, A/S, FOX	*chuA*^*+*^, *yjaA*^*+*^,*TspE4*^*+*^, *FosA3*^*+*^
51	B2	MEM, LEV, TE, CZ, CAZ, N, FOX	*chuA*^*+*^, *yjaA*^*+*^,*TspE4*^*+*^, *FosA3*^*+*^, *CTX-M*^*+*^
57	B2	MEM, CZ, CAZ, FOX	*chuA*^*+*^, *yjaA*^*+*^,*TspE4*^*+*^, *FosC2*^*+*^
60	B2	LEV, TE, CZ, N	*chuA*^*+*^, *yjaA*^*+*^,*TspE4*^*+*^, *FosC2*^*+*^
93	D	LEV,TE, CZ, CAZ, N, A/S, FOX	*arpA*^*+*^, *chuA*^*+*^, *FosA3*^*+*^
30	D	LEV, TE, CZ, CAZ, N	*arpA*^*+*^, *chuA*^*+*^, *FosA*^*+*^

UPEC, Uropathogenic *E. coli*; CZ, Cefazolin;A/S, Ampicillin-Sulbactam; FOX, Cefoxitin; CAZ, Ceftazidime; LEV, Levofloxacin; N, Nitrofurantoin; TE, Tetracycline; MEM, Meropenem

**Table 6:** Frequency of the different fos genes and CTX-M gene among fosfomycin resistant *E. coli* phylogenic groups

Group	*fos*A	*fos*A3	*fos*A4	*fos*A5	*fos*C2	CTX-M
B2	0	2	0	0	2	1
D	1	1	0	0	0	0
E	7	8	1	1	2	7
Total	8	11	1	1	4	8

## Discussion

In this study 241 urine samples obtained from children suffering from UTIs, 151(62.7%) samples obtained from females and 90 (37.3) from males.170 samples were confirmed as positive cultures; 96 of these cultures were confirmed to be *E. coli*. Fosfomycin resistance was reported in 38.5% of *E. coli* isolates by agar dilution method. Phylogenetic analysis in this study showed that UPEC isolates mainly belong to group B2 followed by group E and D. Previous studies have shown that female gender, history of prolonged antibiotics administration, family history of UTI and enuresis were important risk factors for UTI [19, 20]. Thus, the high frequency of these risk factors in children with UTI found in our study is consistent with these reports. In this study, *E. coli* was the predominant etiological agent (56.5%) of UTI in children and the distribution of UPEC in the same age group is similar to that previously reported [21, 22].

Fosfomycin was reintroduced as an option for the treatment of UTI in children. There are few epidemiological, clinical or molecular data on *E. coli* strains resistant to fosfomycin isolated from UTIs. In this study, we investigated the occurrence and molecular features of all fosfomycin-resistant *E. coli* isolated from children. The strains in our study displayed a high rate of resistance to fosfomycin of about 38.5%, which is higher than that previously reported in Spain, Europe, Asia, United States, South Africa and Pakistan. In Spain certain studies reported that, fosfomycin- resistance among *E. coli* isolated from urine samples of adult patients were 2.7% and 3.8%, respectively; however, this rate increased to 14.1% and 21.7% when only ESBL-producers were considered [9, 23]. Also, Garcia-Fulgueiras et al. and Rodríguez-Lozano et al. in Spain reported that fosfomycin- resistance among microorganisms that cause urinary tract infections in pediatric patients was 4% and 14.5%, respectively [8, 24]. A total of 1225 ESBL-producing *E. coli* isolated from different samples and different age groups were investigated for fosfomycin resistance in Switzerland, Europe by Mueller et al. Seventeen isolates turned to be positive according to the results of the agar dilution method and rapid fosfomycin/*E. coli* NP test [25]. Mosime et al. in South Africa reported that, amongst all the *E. coli* isolates (n = 138) collected from antenatal clinics, fosfomycin resistance was detected in 2.2% of these isolates [26]. Ali et al. in Pakistan reported 10% resistance to fosfomycin in UPEC isolated from Patients with different age groups[27]. The difference in Fosfomycin resistance rates could be due to different sample size and different geographical distribution.

Following our result, Abu El-Wafa and Abouwarda in Egypt reported 24.1% resistance to fosfomycin in UPEC isolated from Patients with different age groups [28].

Oteo et al. reported that fosfomycin resistance among ESBL-producing *E. coli* increased from 2.2% to 2003 to 21.7% in 2008 with the increased use of fosfomycin by 50% (23). Moreover, Lee and Le [29] have reported that the increased use of fosfomycin has been associated with increased resistance.

The overall resistance of UPEC isolates to different antibiotics was; Cefazolin (92.7%), Ampicillin-Sulbactam (57.3%), Cefoxitin (63.5%), Ceftazidime (71.8%), Levofloxacin (81.3%), Nitrofurantoin (76%), Tetracycline (82.3%), and Meropenem (39.6%). The high antibiotic resistance rates and MDR pattern (71.8%) of UPEC isolates found in our study is consistent with previous reports from Egypt whether UPEC isolates were collected from children or from different age groups [30–33]. This may be due to non-indicated, random and excessive use of these antibiotics. Thus the guidelines for antibiotics description must be strictly applied in our country. Also, improving the public knowledge and changing their attitudes towards antibiotic use will be a crucial strategy to maintain antibiotic effectiveness.

Most of the fosfomycin-resistant strains studied in this work showed a high resistant rate to different antibiotics and 85.7% of these isolates were MDR. The resistance rates for cefazolin, cefoxitin, and ceftazidime were significantly higher in fosfomycin-resistant isolates than in fosfomycin-susceptible isolates (P-value < 0.05). Loras et al. and Ya Li et al. also reported that antimicrobial resistance to different antibiotics was significantly higher in fosfomycin-resistant isolates than in fosfomycin-susceptible isolates [9, 34].

In this study, history of prolonged antibiotics administration and history of recurrent UTI were reported as important risk factors for fosfomycin resistance and MDR. Thus, these data suggest certain possible points of intervention to overcome antibiotic resistance such as: restricted administration of antibiotics, optimize antibiotic use among children with recurrent UTI, empirical antibiotics administration for treatment of UTI among children with previous prolonged antibiotic use.

In fosfomycin-resistant strains, phosphonoformate test and PCR for fos genes were performed to detect plasmid-mediated fosfomycin-resistant isolates, followed by classification by phylogenetic grouping. To the best of our knowledge, the present work is the first investigation of the prevalence and phylogenetic grouping of fosfomycin-resistant UPEC in Egypt.

In this study and according to the new Clermont phylotyping method, group B2 (41.7%) was the predominant group among UPEC isolates, followed by E (33.3%) and D (19.8%) groups. Our findings are in accordance with other studies in Egypt [30, 35] and worldwide [2, 36–38] where it was found that virulent extra intestinal *E. coli* strains belonged typically to group B2 followed by group D or E. Most of our fosfomycin-resistant UPEC strains belonged to phylogenetic group E, 23/37 (62.2%) followed by B2 (21.6%) and D (13.5%) groups. On the other hand, Group B2 was the predominant phylogenetic group in the fosfomycin-susceptible isolates, 32/59 (54.2%). This indicates that group E may contribute more to fosfomycin resistance than other phylogroups. Another study carried by Loras et al. in Spain reported that; most of the fosfomycin resistant UPEC belonged to phylogenetic group B2 (23/39; 59%), followed by: E (7/39; 18%), A (5/39; 13%), B1 (2/ 39; 5%) and C (2/39; 5%) groups [9]. This difference may be due to different strains, sample sizes, and geographical distribution.

The phylogroup E strains possess 4331 to 5440 genes with a core genome of 2771 genes and a pangenome of 33 722 genes. The distribution of these genes among the strains shows an asymmetric U-shaped distribution. E phylogenetic strains have the largest genomes of the species, partly explained by the presence of mobile genetic elements. Sixty-eight lineages were delineated, some of them exhibiting extra-intestinal virulence genes and being virulent in the mouse sepsis model. Some diarrheogenic *E. coli* strains are found in this phylogroup and except for the EHEC lineages and the reference EPEC, EIEC and ETEC strains, very few strains possess intestinal virulence genes [39].

Out of 37 fosfomycin-resistant *E. coli* isolates, 23 (62.2%) were phosphonoformate test positive, and the tested *fos* genes were detected in 21 isolates of them. The predominant *fos* gene in this study was *fosA3*, which was found in 11 isolates. It has been previously reported that *fosA3* is the most prevalent plasmid-mediated fosfomycin-resistant gene in *E. coli* isolates, which is usually located in conjugative plasmids also carrying CTX-M-type ESBL-encoding genes [40–42][40, 41 and 42]. Thus, the high prevalence of *fosA3* gene found in our study is consistent with these reports. Moreover, all but 3 of the 11 fosA3-positive isolates in our study were also positive for CTX-M gene, confirming the high degree of association between the two resistance determinants. The small fosfomycin resistant sample set complicated the interpretation of our results. Furthermore, we could only base our findings on the selected resistance genes investigated in this study. Although the study tested only 96 UPEC isolates and all the isolates were from the same region, the study provided some indication of the fosfomycin resistance patterns and genes among UPEC phylogenetic groups in our locality. Further studies on UPEC phylogenetic groups and fosfomycin resistance patterns with large sample size from different regions are recommended to understand the characterization and the epidemiology of this isolates. Future studies on this sample set could use whole genome sequencing to describe other potential fosfomycin resistance mechanisms.

## Conclusion

This study showed that the prevalence of fosfomycin-resistant UPEC start to increase with the increased reuse of fosfomycin antibiotic as a result of high resistance to other antibiotic classes. In this study we detected the presence of different variants of plasmid-mediated *fos* genes in fosfomycin- resistant *E. coli* strains. As far as we know, this is the first description of the plasmid-mediated fosfomycin-resistant UPEC in Egypt. Acquisition of the *fosA* genes by successful *E. coli* clones and efficient resistance plasmid vectors may restrict the usefulness of fosfomycin in MDR infections. Fosfomycin should be used carefully and the prevalence of *fosA* and other *fos* genes monitored closely. This study shows that, in our setting fosfomycin- resistant UPEC strains isolated from children belongs mainly to phylogenetic group E. Alteration in the phylogenetic types are important in identifying novel groups of emerging bacteria that are better recognized as a result of this analysis.

## Data Availability

All data generated or analyzed during this study are included in this article.
